# Effects of intra- and inter-day temperature change on acute upper respiratory infections among college students, assessments of three temperature change indicators

**DOI:** 10.3389/fpubh.2024.1406415

**Published:** 2024-08-23

**Authors:** Feng Jiang, Rensong Wang, Yongli Yang, Xiaocan Jia, Leying Ma, Mengyang Yuan, Kangkang Liu, Junzhe Bao

**Affiliations:** ^1^Department of Disease Prevention and Control, Zhengzhou University Hospital, Zhengzhou University, Zhengzhou, Henan, China; ^2^Department of Emergency, Shanghai Fengxian District Medical Emergency Center, Shanghai, China; ^3^Department of Biostatistics and Epidemiology, College of Public Health, Zhengzhou University, Zhengzhou, Henan, China; ^4^Department of Research Center for Medicine, The Eighth Affiliated Hospital, Sun Yat-Sen University, Shenzhen, China

**Keywords:** DTR, TV, TCN, acute upper respiratory infection, attributable fractions

## Abstract

**Background:**

Acute upper respiratory infection (AURI) is a significant disease affecting all age groups worldwide. The differences in the impacts of different temperature change indicators, such as diurnal temperature range (DTR), temperature variation (TV), and temperature change between neighboring days (TCN), on AURI morbidity, are not clear.

**Methods:**

We collected data on 87,186 AURI patients during 2014–2019 in Zhengzhou. Distributed lag non-linear model was adopted to examine the effects of different temperature change indicators on AURI. We calculated and compared the attributable fractions (AF) of AURI morbidity caused by various indicators. We used stratified analysis to investigate the modification effects of season and gender.

**Results:**

With the increase in DTR and TV, the risk of AURI tended to increase; the corresponding AF values (95% eCI) higher than the references (5% position of the DTR or TV distribution) were 24.26% (15.46%, 32.05%), 23.10% (15.59%, 29.20%), and 19.24% (13.90%, 24.63%) for DTR, TV_0 − 1_, and TV_0 − 7_, respectively. The harmful effects of TCN on AURI mainly occurred when the temperature dropped (TCN < 0), and the AF value of TCN below the reference (0°C) was 3.42% (1.60%, 5.14%). The harm of DTR and TV were statistically significant in spring, autumn and winter, but not in summer, while the harm of TCN mainly occurred in winter. Three indicators have statistically significant effects on both males and females.

**Conclusions:**

High DTR and TV may induce AURI morbidity, while the harm of TCN occurs when the temperature drops. The impacts of DTR and TV on AURI are higher than that of TCN, and the impact of few-day TV is higher than that of multi-day TV. The adverse effects of DTR and TV are significant except in summer, while the hazards of TCN mainly occur in winter.

## 1 Introduction

Acute upper respiratory infection (AURI) is a common disease of all ages worldwide, with symptoms including stuffy nose, cough, fever, malaise, and sore throat (1, 2). In 2017, there were 17.1 (95% UI 15.3 to 19.2) billion AURI incident cases worldwide, which ranked the first leading cause of incident cases (3). AURI is rarely fatal but causes significant morbidity and economic burden. The physical condition of college students is better than that of children and the older adult, but they are also easily threatened by AURI, affecting their regular learning and life (4).

In the context of climate change, the impact of adverse temperature conditions (such as cold and heat) on population health has received extensive attention (5, 6). Most current research is on the effects of high and low temperatures on cardiovascular and cerebrovascular diseases and respiratory diseases (5, 7–9). In addition to the hazards of high and low temperatures, large changes in short-term temperature will also cause harm to the health of the population (10, 11).

As the three most commonly used indicators to reflect temperature changes, the diurnal temperature range (DTR) could reflect the intra-day temperature change, the temperature variation (TV) of 2 days or more could reflect the intra- and inter-day temperature change (12), and the temperature change between neighboring days (TCN) could reflect inter-day temperature changes. Compared with DTR and TV, TCN not only reflects the degree of temperature change but also the direction of temperature change, such as the increase or decrease of temperature (13). DTR and TCN were two typical indicators of temperature variability, but some studies have found that the innovation indicator (TV) was superior in estimating ambient temperature variability (12). These three indicators, especially TV, could accurately represent heat and cold waves, reflecting the diurnal cycle of ambient temperature and air circulation (14). A study from Wuhan University reported that DTR was associated with an increased risk of AURI (15). However, few studies have comprehensively compared the effects of different intra- and inter-day temperature change indicators on population health, especially on AURI.

Therefore, this study aimed to clarify the short-term effects of DTR, TV, and TCN on the outpatient visits of AURI. We furtherly compare their effects by calculating their attributable fractions (AF) for AURI. To our best knowledge, this is the first study on this topic in China.

## 2 Materials and methods

### 2.1 Data source

Zhengzhou is located in the center of China, with a longitude of 112°42′-114°13′ East and a latitude of 34°16′-34°58′ North (Supplementary Figure S1). Zhengzhou has a warm, temperate sub-humid monsoon climate. Zhengzhou has four distinct seasons: hot summer and cold winter; spring and autumn temperatures are relatively mild, but the temperature changes frequently in these seasons.

The data of outpatient visits were collected from the Hospital of Zhengzhou University, a university-level hospital affiliated with Zhengzhou University. Zhengzhou University is a famous university with about 70,000 students, making it the university with the most significant number of students in China. As the nearest and designated hospital for university students' medical insurance, it is the first choice for college students of Zhengzhou University to seek medical support and public health services, especially for less severe illnesses like AURI.

The dataset ranged from January 1, 2014, to December 31, 2019, including patients' gender, age, occupation, visit date, and diagnosis. We screen AURI patients based on diagnosis and student patients based on occupation. Meteorological data and air pollution data were collected from the National Meteorological Data Sharing Platform and National Urban Air Quality Realtime Publishing Platform; for detailed information, please refer to our previously published papers (6).

### 2.2 Data analysis

Distributed lag non-linear model (dlnm) and Poisson regression were adopted to examine the effects of different temperature change indicators on AURI. Possible confounders were included in the model, including relative humidity, temperature, air pollutants (in the main model, PM_2.5_ were selected to represent air pollution), seasonal fluctuations, day of the week, public holidays, and vacation. We adopted a natural cubic spline with 3 degrees of freedom (df) for temperature and relative humidity. We used a natural cubic spline with 7 df per year for time to control the influence of long-term trend as well as seasonal fluctuations. We set day of the week, public holidays, and vacations as categorical variables. Based on the Akaike information criterion for the quasi-Poisson models, the value of df for each variable was set (Supplementary Table S6). The basic model was presented below:


$$\begin{array}{l} {\;Y}_{\text{t}}\sim quasi-Poisson\;\left(\mu_{\text{t}}\right)\\ Log\left(\mu_{\text{t}}\right)=\alpha +Cb.TC_{\text{t}}+ns\left(RH_{\text{t}},3\right)+ns\left(Tmean_{\text{t}},3\right)\\ \;+AP_{\text{t}}+Vacation+Holiday+ns\left(time,7\times 6\right)+DOW \end{array}$$

Where μ_t_ denoted the number of AURI patient visits among college students on day t of the study; α represented the intercept; Cb.TC_t_ referred to the cross-basis matrices of temperature change indicators; ns was a natural cubic spline function; RH_t_ and Tmean_t_ were relative humidity and mean temperature, respectively; AP_t_ was used to control the effects of air pollutants; time with 7 df per year was used to control seasonal and long-term trends; Holiday and Vacation for controlling public holidays and summer/winter vacation effects; DOW meant days per week.

Within the cross-basis of temperature change indicators, a natural cubic spline with 3 df was adopted, and the maximum lag days was set as 7. As for DTR and TV, the value corresponding to the 5% position of the temperature change distribution was set as the reference value. As for TCN, 0°C was set as reference. We estimated the risk of AURI attributable to high temperature change (for DTR and TV, higher than the references) or temperature drops (for TCN, TCN < 0) and summed the number of college student AURI due to temperature changes and divided by the total number of college student AUTIs in the dataset to derive the risk of AURI due to temperature changes (AF) (5, 16). In addition, we adopted Monte Carlo simulations to calculate empirica CI (eCI). DTR was calculated as the difference between the maximum and minimum temperature on the same day (17). TV was calculated as the standard deviation (SD) of the minimum and maximum temperatures during the exposure days. For example, TV_0 − 1_ was the SD of the minimum and maximum temperature of the current day as well as the minimum and maximum temperature of the previous day (12). TCN was calculated as the difference in mean temperature between the current day and the previous day (18).

The formulas were as follows:


$$\begin{array}{r} DTR=Tmax-Tmin\\ TV_{\text{0}-\text{t}}=SD(Tmax\_day0,Tmin\_day0,Tmax\_day1,\\ Tmin\_day1\ldots Tmax\_dayt,\;Tmin\_dayt)\\ TCN=Tmean_{\text{t}}-Tmean_{\text{t-1}} \end{array}$$

In order to identify the high-risk periods and vulnerable populations, we took subgroup analyses based on season and gender. We also tested whether there were differences between genders using Z-tests (19).

### 2.3 Sensitivity analysis

We changed df values for the time term, the lag days of temperature change indicators, different reference values of temperature change indicators and controlled for other air pollutants (such as CO and O_3_) besides PM_2.5_, so as to test the robust of our analysis. In addition, given the potential confounding of influenza, we defined influenza as the number of outpatient visits in a week that was greater than the 75th percentile of the total number of weekly outpatient visits in that year, and treated it as a dummy variable to control for in the model for the sensitivity analysis (4).

R software (version 3.4.0) was adopted in the data analysis. Two-tailed *p* < 0.05 were considered statistically significant.

## 3 Results

### 3.1 Descriptive analysis

There were 87,186 AURI patients during 2014–2019, with slightly more women (45,352, 52%) than men (41,834, 48%) (Table 1). The daily mean temperature was 16.5 (10.0)°C, the daily mean DTR was 9.8 (4.1) °C, and the daily mean PM_2.5_ concentration was 75.1 (56.1) ug/m^3^ (Supplementary Table S1).

**Table 1:** Distributions of daily outpatient visits for AURI.

**Variables**	**Min**	**P25**	**P50**	**P75**	**Max**	**Mean**	**SD**
Daily patients (*n* = 87,186)	0.0	17.0	36.0	55.0	210.0	39.8	29.9
**Gender**							
Male (*n* = 41,834, 48%)	0.0	8.0	17.0	26.0	103.0	19.1	14.9
Female (*n* = 45,352, 52%)	0.0	8.0	18.0	29.0	107.0	20.7	15.9
**Season**							
Spring (*n* = 27,026, 31%)	5.0	32.0	44.0	63.0	146.0	49.0	24.3
Summer (*n* = 11,793, 14%)	0.0	10.0	16.0	28.0	89.0	21.4	16.1
Autumn (*n* = 27,042, 31%)	4.0	30.0	43.0	65.0	157.0	49.5	27.9
Winter (*n* = 21,325, 24%)	0.0	8.0	35.0	57.0	210.0	39.4	38.2

Min and Max represent minimum and maximum values; P25, P50, and P75 represent the 25th percentile, median, and 75th percentile; Mean and SD represent mean and standard deviation.

**Figure 1:**
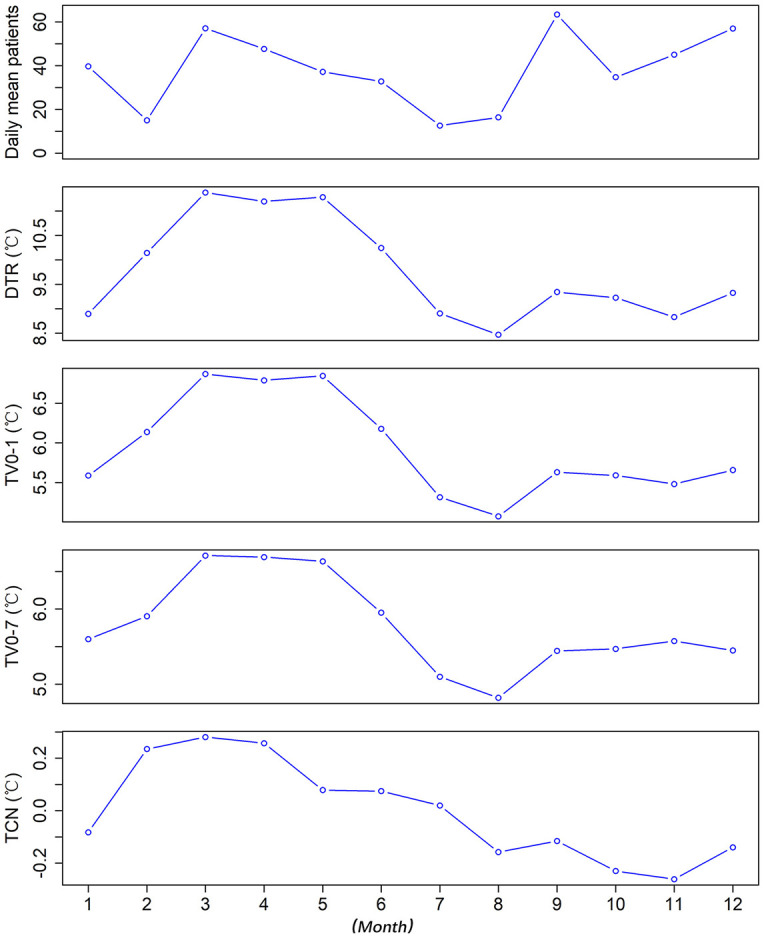
The distribution of daily mean values of AURI patients, DTR, TV_0 − 1_, TV_0 − 7_, and TCN in different months^*^. ^*^DTR was the difference between the maximum and minimum temperature on the same day. TV was the standard deviation of the minimum and maximum temperatures during the exposure days. TCN was the difference in mean temperature between the current day and the previous day.

The daily mean AURI was higher in March and September. DTR and TV were higher in March and April. TCN was high in March and April while low in October and November (Figure 1).

### 3.2 Associations between intra- and inter-day temperature change and AURI

**Figure 2:**
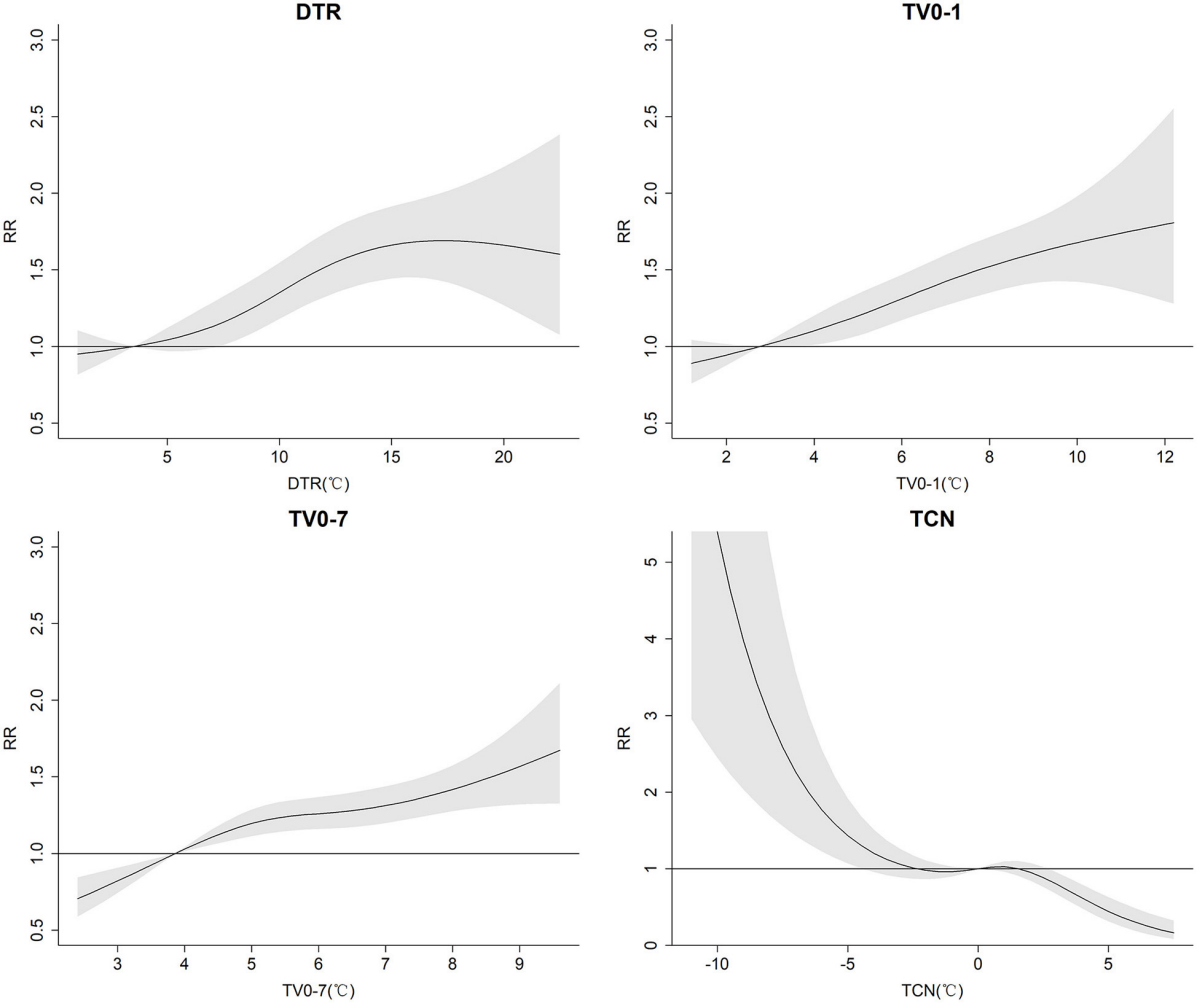
The cumulative exposure-response associations between DTR, TV_0 − 1_, TV_0 − 7_, TCN, and AURI in college students of Zhengzhou*. *DTR was the difference between the maximum and minimum temperature on the same day. TV was the standard deviation of the minimum and maximum temperatures during the exposure days. TCN was the difference in mean temperature between the current day and the previous day.

With the increase of DTR and TV, the risk of AURI tended to increase. When the DTR reached 15°C, the risk of AURI was stable and no longer increased with the increase of DTR, while TV_0 − 1_ and TV_0 − 7_ did not show a clear threshold. The harmful effects of TCN on AURI mainly occurred when the temperature dropped; the risk of AURI increased the more the temperature fell as TCN fell below −3°C, whereas a rise in temperature had a protective effect once TCN exceeded 2°C (Figure 2).

**Figure 3:**
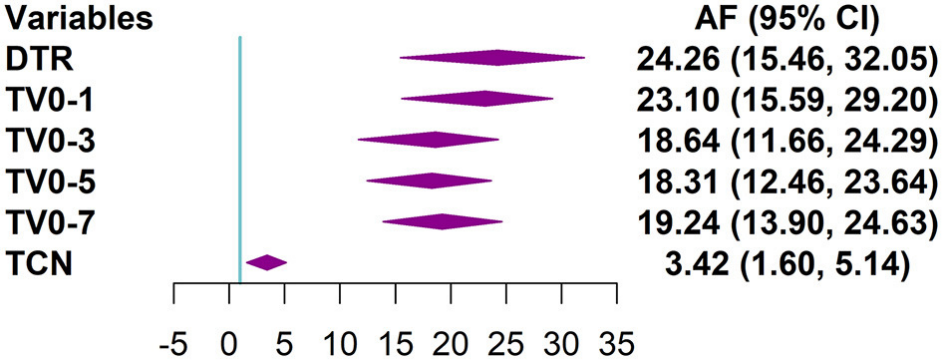
Forest plot of the distribution of the attributable fractions of AURI corresponding to DTR, different TV index, and TCN*. *DTR was the difference between the maximum and minimum temperature on the same day. TV was the standard deviation of the minimum and maximum temperatures during the exposure days. TCN was the difference in mean temperature between the current day and the previous day. For TCN, only show AF (95% CI) caused by TCN <0.

The harmful effects of DTR, TV, and TCN were mainly maintained within seven days, and the effects on the day of adverse temperature changes occurred were significant (Supplementary Figure S2). Regarding the morbidity of AURI, the AF (95% CI) for DTR was 24.26 (15.46, 32.05). The AF (95% CI) for TV_0 − 1_ was 23.10 (15.59, 29.20), higher than that for TV_0 − 3_, TV_0 − 5_, and TV_0 − 7_, which were 18.64 (11.66, 24.29), 18.31 (12.46, 23.64) and 19.24 (13.90, 24.63). They are all higher than TCN, which was 3.42 (1.60, 5.14) (Figure 3).

### 3.3 Subgroup analysis and sensitivity analyses

Adverse effects of DTR were significant in spring, autumn, and winter, with AF (95% CI) of 22.45 (14.27, 30.00), 11.36 (1.72, 19.69) and 17.62 (2.92, 30.28), respectively; whereas adverse effects were not significant in summer. Adverse effects of TV were similarly significant in spring, autumn, and winter, with AF (95% CI) 10.31 (3.37, 16.83), 13.88 (7.11, 19.91), and 23.65 (14.53, 30.90), respectively, whereas adverse effects were not significant in the summer months. Adverse effects of TCN were mainly in the winter months, with an AF (95% CI) of 17.88 (7.72, 26.21).

**Figure 4:**
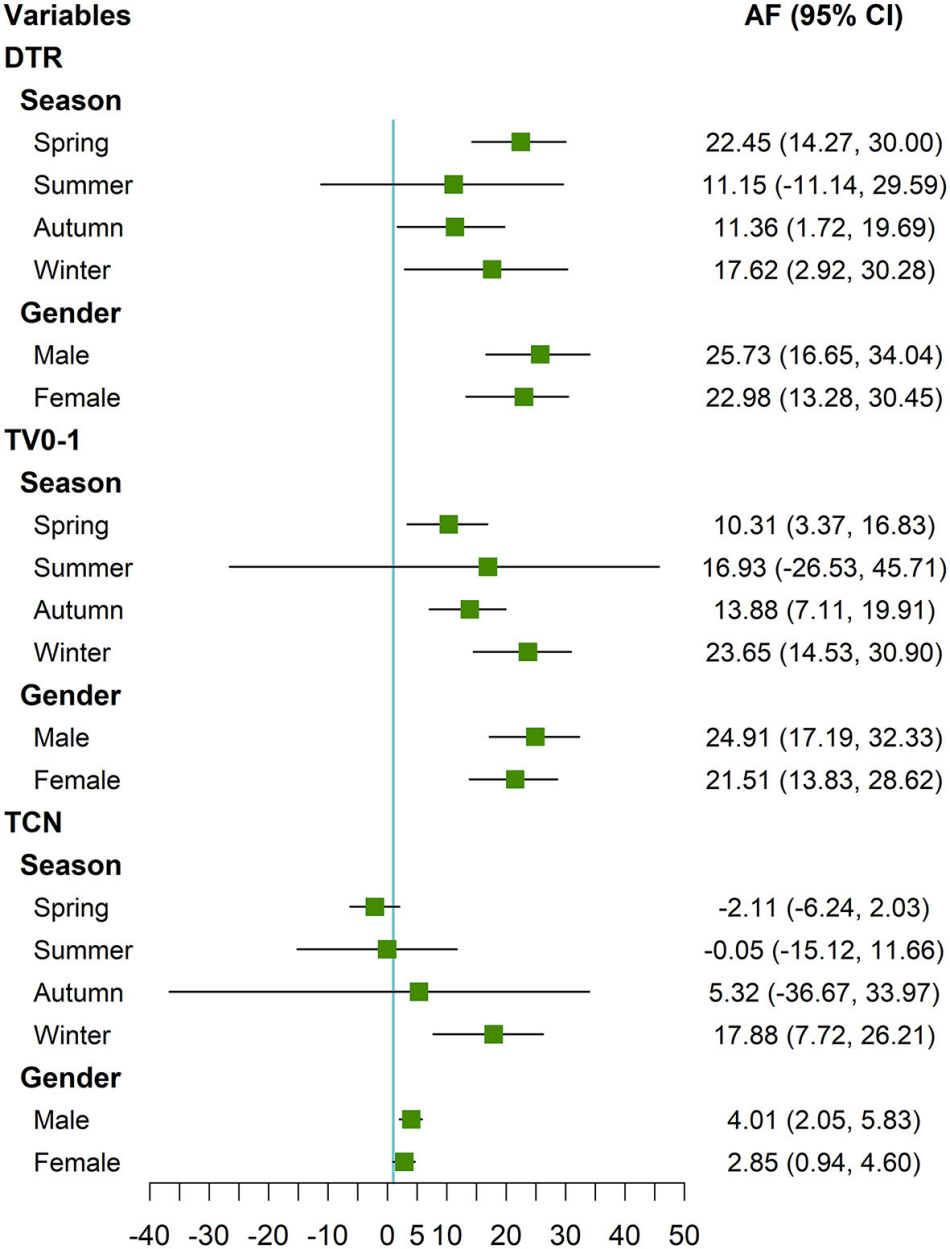
Forest plot of the distribution of the attributable fractions of AURI corresponding to DTR, TV_0 − 1_, and TCN in different seasons and gender*. *DTR was the difference between the maximum and minimum temperature on the same day. TV was the standard deviation of the minimum and maximum temperatures during the exposure days. TCN was the difference in mean temperature between the current day and the previous day. For TCN, only show AF (95% CI) caused by TCN <0.

The harmful effects of DTR, TV, and TCN on males were statistically significant, with AF (95% CI) of 25.73 (16.65, 34.04), 24.91 (17.19, 32.33), and 4.01 (2.05, 5.83), respectively. And on females, DTR, TV, and TCN were also statistically significant with AF (95% CI) of 22.98 (13.28, 30.45), 21.51 (13.83, 28.62), and 2.85 (0.94, 4.60). The AF values were slightly higher in males than females (Figure 4). However, these differences were not statistically significant (*P* > 0.05) (Supplementary Table S6).

After using different df values for the time term, using different lag times, using different reference values in the model, and controlling for different air pollutants or influenza, the corresponding AF values did not change much, indicating that the models were robust (Supplementary Tables S2–5).

## 4 Discussion

In this study, we found that the risk of AURI morbidity tended to increase with the increase of DTR and TV, and the harmful effects of TCN mainly occurred when the temperature decreased. DTR and TV had greater effects on AURI than TCN, and short-term TV (such as TV_0 − 1_) had greater effects than multi-day TV (such as TV_0 − 7_). The harmful effects of DTR and TV were significant in spring, autumn and winter, but not in summer, while the harmful effects of TCN mainly occurred in winter. The harmful effects of all these indicators were statistically significant for both men and women.

Several previous studies have also found that increased DTR and TV might lead to an increased risk of respiratory diseases. A study in Wuhan found that 1°C increase in DTR would lead to a 1.08% (95% CI: 0.22, 1.95) increase in the daily number of outpatient visits of all college students for AURI at a lag of 0–6 days (4). A study in Shanghai found that 1°C increase in DTR would lead to a 1.60 % (0.62, 2.58) increase in emergency-room visits for respiratory tract infection at a lag of 0–5 days (20). A study in 21 cities in South China found that the increase in DTR was associated with the hospitalization of respiratory diseases (17). Studies in Dhaka and Brazil found that the increase in TV was associated with respiratory disease morbidity. They found that TV was most strongly associated with respiratory admissions compared with cardiovascular disease and other diseases (21, 22).

Some potential mechanisms could be used to explain the harmful effects of DTR and TV on the respiratory system. (1) Temperature change might influence the function of respiratory epithelium, then affect the defense of the respiratory system, nasal responses, and airway mucociliary clearance (23). (2) Significant temperature change might promote the transmission and reproduction of bacteria and viruses, then induce the morbidity of respiratory diseases (24). (3) People may not add or remove clothing in time when they encounter sudden temperature changes, resulting in colds, weakened immunity, increased respiratory load, and bacterial or viral infections (22). These factors could easily induce respiratory diseases.

This study revealed that the adverse effects of TCN occurred when the temperature dropped. A study in Brisbane found that a sharp drop in temperature could induce the morbidity of childhood pneumonia. In contrast, the temperature increase between neighboring days did not adversely affect childhood pneumonia (25). Similarly, a study in Hefei also found that a significant temperature drop between two neighboring days had adverse effects on childhood allergic rhinitis and tuberculosis admissions (26, 27). On the other hand, a study in United States found that negative TCN (temperature decrease from the previous day) was associated with reduced total non-accidental mortality as well as respiratory mortality, while positive TCN (temperature increase) elevated the risk of mortality (13). A study in Maanshan of China also found that positive TCN was associated with elevated mortality risk from non-accidental and cardiovascular disease (28).

Possible reasons for the differences in the harmful effects of TCN on population health among different studies include (1) the differences in weather conditions, socio-demographic characteristics, and living conditions such as the use of air conditioners (29). (2) The effects of TCN might differ in morbidity and mortality as well as different diseases. Sudden temperature drop might induce the morbidity of acute respiratory infection (25–27) but have insignificant effects or even protective effects on mortality of some other diseases (13, 28).

The AF of DTR and TV on AURI were greater than that of TCN, and the AF of short-term TV was greater than that of multi-day TV. According to the calculation formula, TCN is based on the difference between the daily mean temperatures on neighboring days. Suppose the daily maximum temperature is high and the daily minimum temperature is low on a specific day. In that case, the corresponding daily mean temperature may be neither high nor low, and the corresponding TCN may be neither high nor low. However, the corresponding DTR and TV should be high. According to the previous description, when the temperature fluctuates wildly, it may cause harm to population health. Therefore, TCN may not fully reflect the harmful effects of this situation, resulting in its AF value being lower than DTR and TV. A multi-country study found that different countries had different patterns of TV-mortality associations, TV_0 − 1_ was suitable for hot areas, and TV_0 − 7_ was suitable for moderate areas (12). AURI is an acute disease and may be more susceptible to short-term temperature changes, such as high DTR or TV. Temperature fluctuations over multiple days may prompt people to strengthen their defenses, and the range of TV over multiple days is smaller than that of the neighboring days (Table 1). These factors may cause the AF value of TV_0 − 1_ to be larger than the AF value of TV for multiple days.

The harmful effects of DTR and TV on AURI were statistically significant except in summer, while the harmful effect of TCN mainly occurred in winter. A study in Shanghai found that high DTR have greater impact on childhood asthma in the cold season (30). A study in Hefei found that DTR significantly affected tuberculosis morbidity in spring, while TCN had significant effects in winter (27). A study in Wuhan found that the effect values of DTR in autumn and winter were higher than the other two seasons (4). DTR, TV and TCN had adverse effects on both men and women. It is slightly more harmful for men than for women. However, this difference was not statistically significant (*P* > 0.05). This may be due to the fact that male college students prefer outdoor sports, their self-protection awareness is not as strong as female students, and they were more vulnerable to the effects of adverse climate change (31).

This study has several limitations: (1) The study used meteorological and pollution data from monitoring sites rather than individual exposures, so there may be exposure assessment bias. (2) This study is based on data from a single city, and the findings may not apply to cities with other climatic conditions and socio-economic environments. (3) This study is based on the data of medical visits of college students, and the findings may not apply to other populations, such as the older adult and children. (4) This study was an association study and cannot be used for causal inference. (5) This study is ecological and suffers from the ecological fallacy.

## 5 Conclusions

High DTR and TV may induce the onset of AURI, while the harm of TCN mainly occurs when the temperature drops. The impacts of DTR and TV on AURI are greater than that of TCN, and the impact of few-day TV is higher than that of multi-day TV. The hazards of DTR and TV are significant except in summer, while the hazards of TCN mainly occur in winter. Government organizations and individuals should pay attention to the hazards of temperature changes, and take countermeasures in advance, such as strengthening publicity, adding or removing clothing in time, and reducing outdoor exposure.

## Data Availability

The datasets presented in this article are not readily available because authors have no right to share data. Requests to access the datasets should be directed to baojz@zzu.edu.cn.
