# Supplementary material for: Effects of intra- and inter-day temperature change on acute upper respiratory infections among college students, assessments of three temperature change indicators
Source: Front Public Health. 2024 Aug 23;12:1406415. doi: 10.3389/fpubh.2024.1406415 (PMC11377250; doi:10.3389/fpubh.2024.1406415)
Supplement: Supplementary file 1 [file Data_Sheet_1.docx]

**Supplementary materials**


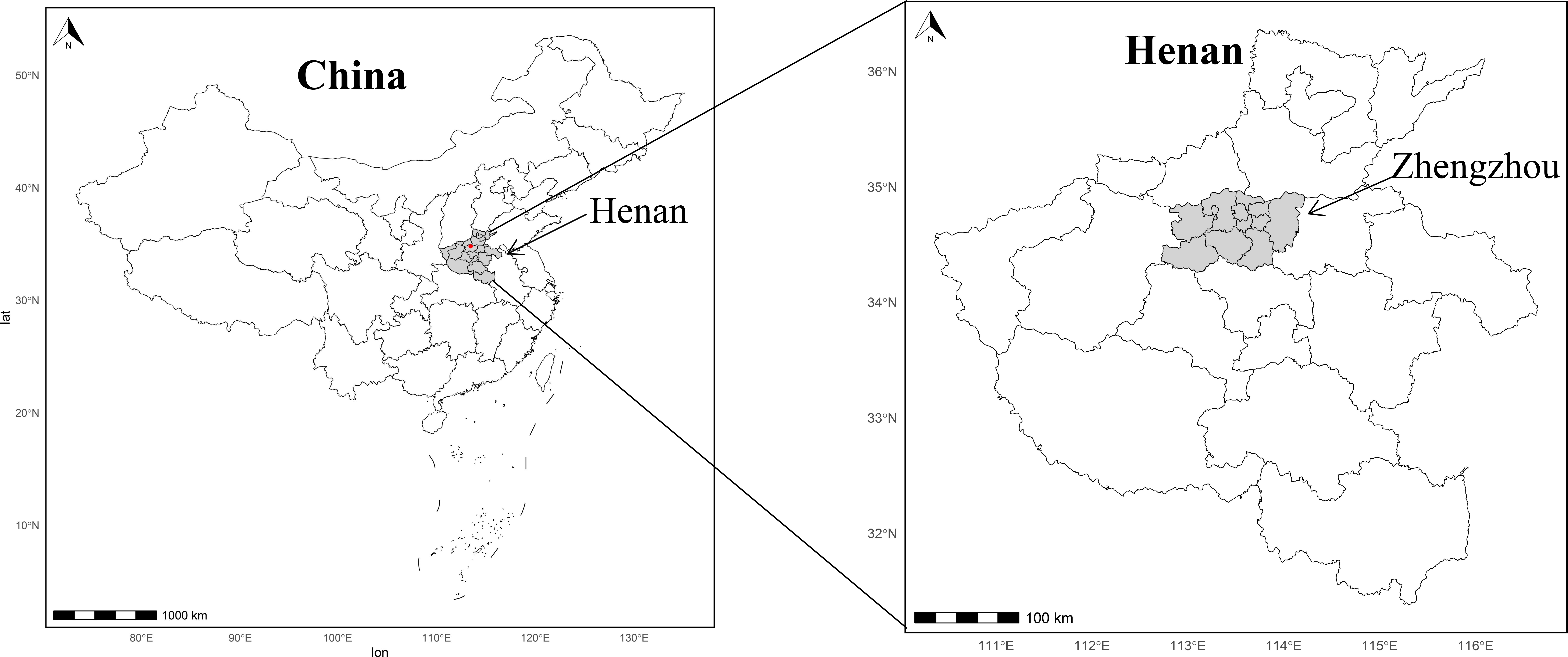


Figure S1. The location of Zhengzhou, which is the capital of Henan Province.

**
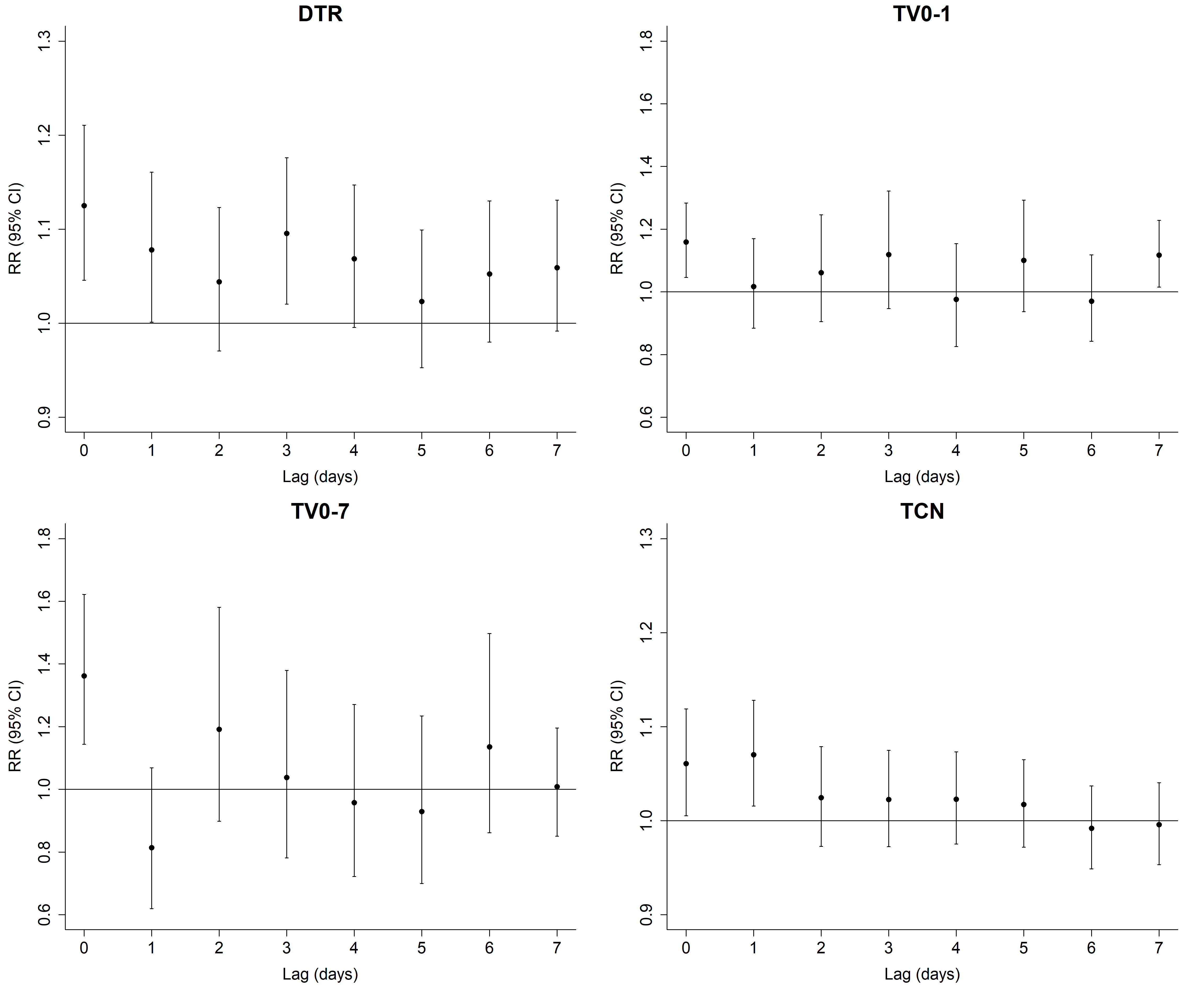
**

Figure S2. The distribution of RR values under different lag times for DTR, TV_0-1_,

TV_0-7_ and TCN^*^.

^*^DTR was the difference between the maximum and minimum temperature on the same day. TV was the standard deviation of the minimum and maximum temperatures during the exposure days. TCN was the difference in mean temperature between the current day and the previous day.

| Table S1. Distributions of daily outpatient visits for meteorological factors, and air pollutants. | | | | | | | |
| --- | --- | --- | --- | --- | --- | --- | --- |
| Variables | Min | P25 | P50 | P75 | Max | Mean | SD |
| Meteorological factors |  |  |  |  |  |  |  |
| Mean temperature (℃) | -5.7 | 7.4 | 17.5 | 25.4 | 34.6 | 16.5 | 10.0 |
| Relative humidity (%) | 13.0 | 46.0 | 60.0 | 74.0 | 100.0 | 59.4 | 18.6 |
| DTR (℃) | 0.6 | 6.7 | 9.5 | 12.7 | 22.6 | 9.8 | 4.1 |
| TV_0-1_ (℃) | 1.0 | 4.6 | 5.8 | 7.3 | 12.3 | 5.9 | 2.0 |
| TV_0-7_ (℃) | 2.3 | 4.9 | 5.7 | 6.6 | 9.8 | 5.8 | 1.2 |
| TCN (℃) | -11.0 | -1.3 | 0.2 | 1.5 | 7.5 | 0.0 | 2.3 |
| Air pollutants |  |  |  |  |  |  |  |
| PM_2.5_ (ug/m^3^) | 7.7 | 38.1 | 57.9 | 93.5 | 527.0 | 75.1 | 56.1 |
| PM_10_ (ug/m^3^) | 16.7 | 82.0 | 119.0 | 174.0 | 604.0 | 137.0 | 75.5 |
| O_3_ (ug/m^3^) | 5.0 | 31.8 | 55.0 | 84.5 | 186.0 | 60.8 | 35.0 |
| CO (mg/m^3^) | 0.4 | 0.9 | 1.2 | 1.6 | 5.5 | 1.3 | 0.6 |
| NO_2_ (ug/m^3^) | 9.1 | 36.0 | 47.9 | 62.8 | 135.0 | 50.7 | 19.1 |
| SO_2_ (ug/m^3^) | 2.1 | 10.6 | 18.9 | 33.0 | 137.0 | 25.0 | 21.0 |

Min and Max represent minimum and maximum values; P25, P50, and P75 represent the 25th percentile, median, and 75th percentile; Mean and SD represent mean and standard deviation.

| Table S2. The distribution of AF values (95% eCI) for AURI caused by DTR, TV_0-1_, and TCN with different df values for the time term in the models. | | | |
| --- | --- | --- | --- |
| Variables | AF (%) | 95% eCI | |
|  |  | Lower limit | Upper limit |
| DTR (df) | |  |  |
| 6 | 23.79 | 15.47 | 32.05 |
| 7 | 24.26 | 15.73 | 32.72 |
| 8 | 23.05 | 15.04 | 30.59 |
| TV_0-1_ (df) | |  |  |
| 6 | 23.38 | 16.04 | 30.34 |
| 7 | 23.10 | 15.31 | 30.51 |
| 8 | 23.65 | 16.40 | 30.51 |
| TCN (df) | |  |  |
| 6 | 3.85 | 2.00 | 5.48 |
| 7 | 3.42 | 1.59 | 5.08 |
| 8 | 3.55 | 1.72 | 5.40 |

| Table S3. The distribution of AF values (95% eCI) for AURI caused by DTR,  TV_0-1_, and TCN with different lag times in the models. | | | |
| --- | --- | --- | --- |
| Variables | AF (%) | 95% eCI | |
|  |  | Lower limit | Upper limit |
| DTR (Lag times) | |  |  |
| 4 | 17.63 | 9.87 | 24.34 |
| 5 | 19.9 | 11.86 | 27.09 |
| 6 | 21.63 | 12.81 | 29.19 |
| 7 | 24.26 | 16.17 | 31.72 |
| 8 | 26.16 | 16.82 | 33.27 |
| 9 | 25.92 | 16.91 | 33.47 |
| TV_0-1_ (Lag times) | |  |  |
| 4 | 16.68 | 10.4 | 22.73 |
| 5 | 18.5 | 11.21 | 24.68 |
| 6 | 21.29 | 14.16 | 27.83 |
| 7 | 23.1 | 15.46 | 29.56 |
| 8 | 23.05 | 15.67 | 30.4 |
| 9 | 23.46 | 15.94 | 30.15 |
| TCN (Lag times) | |  |  |
| 4 | 2.19 | 0.94 | 3.22 |
| 5 | 2.76 | 1.35 | 4.04 |
| 6 | 2.98 | 1.37 | 4.55 |
| 7 | 3.42 | 1.69 | 5.06 |
| 8 | 3.06 | 1.18 | 5.02 |
| 9 | 2.38 | 0.13 | 4.55 |

| Table S4. The distribution of AF values (95% eCI) for AURI caused by DTR,  TV_0-1_, and TCN with different air pollutants or influenza in the models. | | | |
| --- | --- | --- | --- |
| Variables | AF (%) | 95% eCI | |
|  |  | Lower limit | Upper limit |
| DTR |  |  |  |
| +PM_2.5_ | 24.26 | 15.64 | 32.15 |
| +PM_10_ | 24.20 | 15.63 | 31.55 |
| +O_3_ | 25.35 | 17.25 | 32.85 |
| +CO | 24.91 | 15.79 | 32.05 |
| +NO_2_ | 20.90 | 12.39 | 29.00 |
| +SO_2_ | 25.50 | 17.46 | 33.09 |
| + influenza | 18.62 | 11.13 | 25.13 |
| TV_0-1_ |  |  |  |
| +PM_2.5_ | 23.10 | 15.75 | 29.17 |
| +PM_10_ | 22.77 | 15.91 | 29.17 |
| +O_3_ | 23.86 | 17.29 | 30.62 |
| +CO | 23.60 | 16.06 | 30.14 |
| +NO_2_ | 20.64 | 13.00 | 27.14 |
| +SO_2_ | 24.00 | 16.57 | 30.52 |
| + influenza | 16.48 | 9.25 | 22.59 |
| TCN |  |  |  |
| +PM_2.5_ | 3.42 | 1.50 | 5.12 |
| +PM_10_ | 3.39 | 1.56 | 5.12 |
| +O_3_ | 3.40 | 1.59 | 5.11 |
| +CO | 3.26 | 1.48 | 5.00 |
| +NO_2_ | 3.65 | 1.94 | 5.25 |
| +SO_2_ | 3.41 | 1.65 | 5.13 |
| + influenza | 2.33 | 0.63 | 3.72 |

| Table S5 The distribution of AF values (95% eCI) for AURI caused by DTR,  TV_0-1_, with different reference values in the models. | | | |
| --- | --- | --- | --- |
| Variables | AF (%) | 95% eCI | |
|  |  | Lower limit | Upper limit |
| DTR |  |  |  |
| P_4_ | 24.96 | 14.61 | 34.07 |
| **P_5_** | **24.26** | **15.46** | **32.05** |
| P_6_ | 23.54 | 16.54 | 30.46 |
| P_50_ | 8.11 | 4.54 | 11.19 |
| TV_0-1_ |  |  |  |
| P_4_ | 23.77 | 15.64 | 30.98 |
| **P_5_** | **23.10** | **15.59** | **29.20** |
| P_6_ | 21.78 | 14.70 | 27.89 |
| P_50_ | 6.14 | 3.34 | 8.67 |

| Table S6. Distribution of AF values (95% eCI) and P-values of Z-tests for AURIs caused by DTR, TV0-1, and TCN in gender subgroups. | | |
| --- | --- | --- |
| Variables | AF values (95% eCI) | *P* |
| DTR |  |  |
| Male | 25.73(16.65,34.04) | 0.66 |
| Female | 22.98(13.28,30.45) |  |
| TV0-1 |  |  |
| Male | 24.91(17.19,32.33) | 0.55 |
| Female | 21.51(13.83,28.62) |  |
| TCN |  |  |
| Male | 4.01(2.05,5.83) | 0.39 |
| Female | 2.85(0.94,4.60) |  |

| Table S7. Quasi-Akaike information criterion values (QAIC) with the adjustments of the degree of freedom (df) of exposure (temperature, relative humidity) and time in the DTR model | | | |
| --- | --- | --- | --- |
| Variables | df | Q-AIC |  |
| temperature | 2 | 21543.08 |  |
|  | **3** | **21489.58** |  |
|  | 4 | 21503.78 |  |
| relative humidity | 2 | 21494.36 |  |
|  | **3** | **21489.58** |  |
|  | 4 | 21489.86 |  |
| time | 6*6 | 22140.59 |  |
|  | **7*6** | **21489.58** |  |
|  | 8*6 | 21502.61 |  |
